# *TBXA2R* gene variants associated with bleeding

**DOI:** 10.1080/09537104.2018.1499888

**Published:** 2018-08-08

**Authors:** Stuart James Mundell, Andrew Mumford

**Affiliations:** 1 School of Physiology, Pharmacology and Neuroscience, University of Bristol, Bristol, UK; 2 School of Clinical Science and School of Cellular and Molecular Medicine, University of Bristol, Bristol, UK

**Keywords:** Bleeding, GPCR, structure–function, platelets, rare variants, thromboxane receptor TPα

## Abstract

Platelet activity is regulated by a number of surface expressed G protein-coupled receptors (GPCRs) including the α isoform of the thromboxane receptor (TPα receptor). With the advance of genomic technologies, there has been a substantial increase in the identification of naturally occurring rare GPCR variants including in the TBXA2R gene, which encodes the TPα receptor. The study of patients with naturally occurring variants within TBXA2R associated with bleeding and abnormal TPα receptor function has provided a powerful insight in defining the critical role of TPα in thrombus formation. This review will highlight how the identification of these function-disrupting variants of the platelet TPα has contributed important structure-function information about these GPCRs. Further we discuss the potential implications these findings have for understanding the molecular basis of mild platelet based bleeding disorders.

## Abbreviations

GPCR, G protein-coupled receptor; ECL, Extracellular loop; ICL, Intracellular loop; TMD, Transmembrane domain

## 

Platelet activity is regulated by a number of cell surface receptors, including the G protein-coupled α isoform of the thromboxane receptor (TPα receptor). The human TPα receptor was the first human eicosanoid receptor cloned and is a typical Class A rhodopsin-like G protein-coupled receptor (GPCR) (1) with sequence variants in *TBXA2R*, which encodes the TPα receptor implicated in asthma, atopic dermatitis, and, of particular relevance to this review, an autosomal dominant bleeding disorder (2).

The ligand for the TPα receptor, thromboxane A_2_ (TXA_2_) is a product of the oxidative metabolism of arachidonic acid generated by the platelet, thereby acting in an autocrine manner to stimulate TPα receptors (3). Resulting stimulation of G_q/11_ and G_12/13_ heterotrimeric G proteins activates downstream signaling proteins including phospholipase C and RhoA to promote platelet activation. The TXA_2_ pathway is the target for the most widely prescribed antiplatelet drug aspirin, which irreversibly inhibits cyclooxygenase enzymes (COX-1) reducing platelet TXA_2_ generation and TPα receptor stimulation. Despite the efficacy of aspirin, there is still interest in developing direct TPα receptor antagonists in order to preserve the beneficial effects of other prostanoids (such as gastric mucosal protection) that are lost upon global COX inhibition (3,4).

One powerful approach to understanding pathophysiological disease mechanisms is the study of patients with bleeding disorders. For example, analysis of pedigrees with the severe platelet function disorder Glanzmann thrombasthenia assisted discovery of the key platelet integrin αIIbβ3 (5). As with most GPCRs, some insights into TPα receptor biology have emerged from the large number of mutagenesis studies undertaken in order to further understand structure–function relationships of the TPα receptor (3). However, studying the direct impact of *in vitro* mutagenesis on anucleate platelet function *in vivo* is not possible experimentally. The study of patients with naturally occurring variants within *TBXA2R* associated with bleeding and abnormal TPα receptor function has provided a powerful alternative defining the critical role of TPα in thrombus formation.

**Figure 1.**
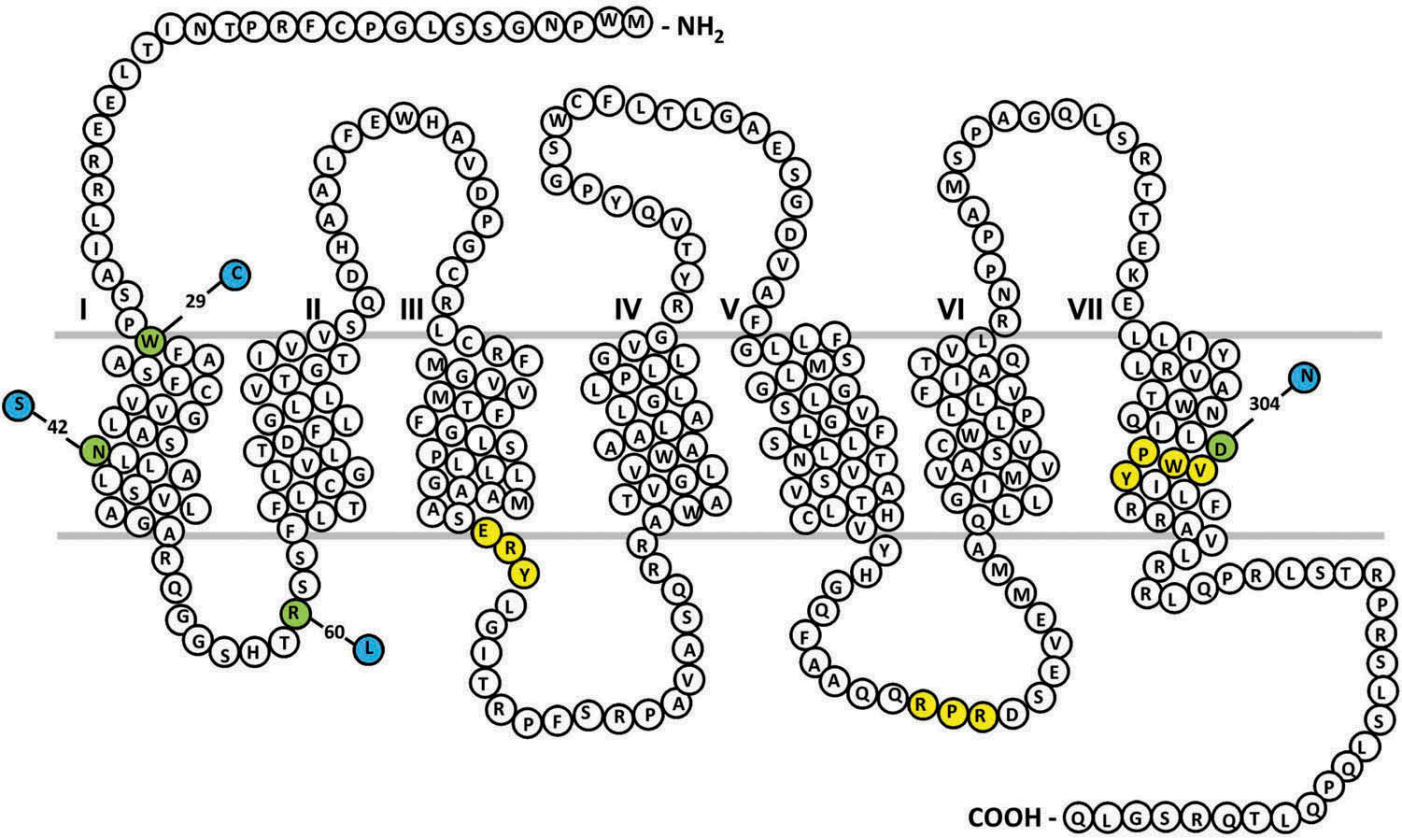
Thromboxane (TP-α) receptor snake plot. Sites of naturally occurring variants found in patients with a bleeding history are highlighted in green. Key amino acid regulatory motifs are highlighted in yellow (specifically RXR ER retention motif; D/NPXXY motif, E/DRY motif).

Thromboxane receptor deficiency (MIM #614009) associated with loss of function *TBXA2R* variants is an autosomal recessive or dominant disorder and has been identified in multiple pedigrees in which some individuals have mild mucocutaneous bleeding symptoms (2). To date, one quantitative defect causing reduced TPα receptor expression (2) and four qualitative defects caused by TPα receptor amino acid substitutions have been reported ((6–9); see Table I and Figure 1).

**Table I.** Variants of *TBXA2R*.

Description	Variation in coding DNA	Inheritance	Region	Defect	Platelet TP receptor phenotype	Reference
Insertion variant causing frameshift	c.167dupG	Heterozygous		Reduced receptor expression	Small and transient platelet aggregation in response to U46619 (2.5 μM) with marked impairment at higher concentration of U46619 (10 μM).	(2)
R60L	c.179G> T	Homozygous or heterozygous	ICL1	Reduced receptor coupling to G_q_	Absence of platelet response to 9,1 1-epithio-1 1,12-methano-TXA_2_ (2 μM).	(9)
D304N	c.190G> A	Heterozygous	TMD7	Reduced ligand binding	Absence of platelet aggregation in response to 0.5 mM AA with reduced level of aggregation to higher AA (1 mM and 1.5 mM) concentrations.	(6)
W29C	c.87G> C	Heterozygous	TMD1	Reduced surface expression	Platelet aggregation in response to AA (1.5 and 2 mM) markedly reduced.	(7)
N42S	c.125A> G	Heterozygous	TMD1	Reduced surface expression	Platelet aggregation and secretion to AA (1 and 1.5 mM) absent.	(8)

The numbering used to describe coding region variants relates to the Ref Seq transcript NM_001060.5. ICL: intracellular loop. TMD: transmembrane domain.

A nucleotide variation which caused loss of TPα receptor expression was first described in a patient with a history of mucocutaneous bleeding (2). Sequence analysis of *TBXA2R* in the patient and her father revealed heterozygosity for a single nucleotide duplication at c.167 (c.167dupG in NM_001060.5) resulting in a frame shift from amino acid 58. Corresponding cell lines studies showed that this nucleotide variation significantly reduced receptor expression.

The first reported qualitative defect in the TPα receptor was caused by a missense *TBXA2R* variant predicting the p.Arg60Leu substitution in the TPα receptor at the start of the first intracellular loop (Figure 1) (9). This variant was first described in a patient with a history of postsurgical bleeding (9), and has since been described in a further pedigree with a history of mild bleeding (10). Platelets from affected individuals show absent or reduced aggregation to the synthetic TXA_2_ analog U46619. In Arg60Leu homozygous patients, this defect in aggregation was accompanied by a reduction in downstream TXA_2_-induced calcium signaling pathways. Interestingly, heterozygous Arg60Leu patients also showed reduced TPα-stimulated platelet aggregation but apparently normal calcium mobilization, suggesting an additional pro-aggregatory effect of TPα receptor activation independent of calcium signaling. The Arg60Leu TPα receptor variant has attenuated receptor responses but comparable ligand-binding affinities and receptor surface expression when compared to wild type (WT) receptor (11). Molecular modeling indicates that Arg60 interacts via hydrogen bonds with Met126 and Arg130 in transmembrane domain (TM)3 and that this interaction is lost when the Arg is substituted for Leu (11). Arg130 is part of the highly conserved D/ERY motif (Figure 1) critical for TPα receptor activation (12). Therefore, in line with previous mutagenesis studies of the ERY motif (12), the Arg60Leu substituted TPα receptor is predicted to be unable to undergo the conformational changes required to promote efficient G protein coupling.

The genotyping and phenotyping of platelets (GAPP) consortium has identified and characterized a series of rare variants in a number of platelet GPCR genes (13,14) including *TBXA2R* (Table I (6–8)). GAPP developed an approach for the rapid identification and characterization of rare genetic variations causing defects within platelet proteins (13). Identification and subsequent characterization of these mutations have significantly enhanced our understanding of structure–function relationships at the TPα receptor

One example was identified in a patient with a history of bruising and prolonged epistaxes since infancy (6). TPα receptor-stimulated platelet activity was reduced in the patient whereas other platelet receptor responses were similar to responses in healthy controls. Sequencing of *TBXA2R* showed a heterozygous c.190G> A variant predicting an Asp304Asn substitution within a highly conserved NPXXY motif in TMD7 (Figure 1). The reduction in TXA2-mediated platelet activation in the patient was due to compromised ligand binding in the Asp304Asn substituted TPα receptor. The NPXXY motif is postulated to weakly stabilize the inactive state of GPCRs and allow the rapid conformational changes required for changes in receptor activation state (15). Why the Asp304Asn substitution causes such a significant decrease in ligand binding at the TPα receptor is unclear. Potentially, this substitution may have a loss of function effect that is unique to the TPα receptor, although it has been postulated that this residue may have a more complex role in stabilizing ligand binding pocket integrity (6).

Two further function-disrupting *TBXA2R* variants predicting amino acid substitutions within TMD1 have also been characterized (Figure 1; (7,8)). Both of these variations reduce TPα receptor expression at the cell surface, suggesting an important role for TMD1 in the regulation of anterograde receptor traffic. The first of these variants, a Trp29Cys substitution, was identified in a patient with abnormal postsurgical bleeding and reduced TPα receptor-mediated platelet activation responses (7). Ligand-binding studies indicated a reduction in both surface receptor expression and ligand-binding affinity with the Trp29Cys variant. There was no change in total receptor expression, but a significant reduction in cell surface expression which was accompanied by a reduced receptor signaling.

The second TMD1 variant, predicting an Asn42Ser substitution, was identified in a patient with significant postoperative and mucocutaneous bleeding (8). As with Trp29Cys, this variant resulted in reduced TPα receptor surface expression and function with the receptor retained intracellularly, in the trans golgi network (TGN)/ER compartment. Asn42, the most conserved residue in class A GPCRs, is therefore required for correct processing and transport of the TPα receptor to the cell surface.

One important observation from this case was that the variant predicting the Asn42Ser substitution was present as a heterozygous trait, indicating that platelets are expected to express both variant and WT TPα receptor. Despite this there was a profound loss of TPα receptor-stimulated platelet function, suggesting a dominant negative effect from the heterozygous variant. Consistent with this, further study revealed that when co-expressed, the Asn42Ser substituted receptor led to intracellular retention of WT receptor. Similar findings have also recently been reported for the platelet P2Y_12_ purinergic receptor in which co-expression of WT and variant receptors can also dramatically reduce P2Y_12_-mediated platelet activation responses, because of a dominant negative effect of the substituted receptor on expression of P2Y_12_ receptor homodimers (16). Importantly, further study revealed both Trp29Cys and Asn42Ser TPα receptor variants were impaired in their ability to dimerize in a recombinant system, with a potential reduction in dimer formation also apparent in platelets taken from the Trp29Cys patient (17). Overall, these data suggest that the impairment of TP dimerization may impact upon platelet aggregation and secretion in response to TP activators *in vivo*.

Beyond these rare population variants, which are associated with strong TPα receptor phenotypes, there are a number of reported common or low-frequency variants that are predicted to alter TXA_2_-mediated platelet responses but which have not been functionally analyzed in patients (18–20). For example, *TBXA2* missense variants observed in population databases that predict Val80Glu and Ala160Thr substitution in the TPα receptor have been expressed in a novel megakaryocyte-based system in an attempt to recapitulate the impact on human platelet responses (18). In this model, the Val80Glu substitution reduced TPα receptor activation whereas the Ala160Thr substitution increased activation responses. It is unknown whether the Ala160Thr substitution confers constitutive activity to the TPα receptor *in vivo*, potentially promoting platelet hyperactivity and conferring increased risk of cardiovascular disease.

In conclusion, the relatively low number of TPα receptor variants identified in patients with abnormal bleeding indicates that these remain rare contributors to bleeding risk even in selected populations. Notably, the presence of heterozygous variants in *TBXA2R* and other GPCR genes does not always correlate with a clinical bleeding phenotype, even though they are consistently associated with abnormal platelet functional responses in diagnostic laboratory tests. For example, although the pedigree index cases with heterozygous variants predicting the Asp304Asn and Trp29Cys TPα receptor substitutions presented with mild bleeding, pedigree members who were also heterozygous for these variants were asymptomatic (6,7). One explanation is that heterozygous loss of function *TBXA2* variants are insufficient alone to cause a clinical bleeding phenotype, but require other mild hemostatic defects in affected patients to manifest as bleeding (21). Indeed, while many hundreds of millions of people take aspirin relatively few have “bleeding disorders” consistent with the lack of bleeding phenotype that can accompany TP receptor mutations and even a complete lack of TP receptor signaling. Studies such as GAPP which use a detailed phenotyping approach combined with targeted genotyping to diagnose platelet function disorders have provided unique insights into key structure–function relationships at the TPα receptor and, in particular, highlighted the role of TMD1 in the regulation of TPα receptor cell surface expression and dimerization. However, in the absence of whole genome sequencing and validation of various non-platelet genes involved in hemostasis, wider speculation on their causal relationship to bleeding should be treated with caution.
